# Antigen-Dependent T Cell Response to Neural Peptides After Human Ischemic Stroke

**DOI:** 10.3389/fncel.2020.00206

**Published:** 2020-07-03

**Authors:** Francesc Miró-Mur, Xabier Urra, Francisca Ruiz-Jaén, Jordi Pedragosa, Ángel Chamorro, Anna M. Planas

**Affiliations:** ^1^Functional Unit of Cerebrovascular Diseases, Hospital Clinic, Barcelona, Spain; ^2^Area of Neuroscience, Institut d’Investigacions Biomèdiques August Pi I Sunyer (IDIBAPS), Barcelona, Spain; ^3^Department of Brain Ischemia and Neurodegeneration, Institut d’Investigacions Biomèdiques de Barcelona (IIBB), Consejo Superior de Investigaciones Científicas (CSIC), Barcelona, Spain

**Keywords:** stroke, T-cell response, antigen-specificity, cytokine production, brain infiltration, brain autopsy

## Abstract

Ischemic stroke causes brain tissue damage and may release central nervous system (CNS)-specific peptides to the periphery. Neural antigen presentation in the lymphoid tissue could prime immune cells and result in adaptive immune response. However, autoimmune responses against neural antigens are not commonly uncovered after stroke. We studied the brain tissue of nine fatal stroke cases and the blood of a cohort of 13 patients and 11 controls. Flow cytometry carried out in three of the brain samples showed CD8 and CD4 T cells in the cerebrospinal fluid (CSF) of the ventricles in the patient deceased 1 day poststroke, T cells with an activated phenotype in the CSF of the patient that died at day 6, and T cells in the ischemic brain tissue in the patient deceased 140 days after stroke onset. Immunohistochemistry showed higher T cell numbers in the core of the lesion of the patient deceased 18 days post-stroke than in the patients deceased from 1 to 5 days post-stroke. In blood samples, we studied whether lymphocytes were primed in the periphery against neural antigens at sequential times (on admission, day 5, and day 90) after stroke. T lymphocytes of stroke patients produced IFN-γ and TNF-α and responded to MBP peptides by increasing their production of TNF-α and IL-10 at admission, but not at later time points. In contrast, IL-4 producing T cells showed progressive increases. Higher percentages of TNF-α producing T lymphocytes at admission were independently associated with poorer outcomes at 90 days. However, we did not detect T cell responses to neural-antigen stimulation 90 days post-stroke. Altogether the results suggest acute T cell priming in the periphery in acute stroke, T cell trafficking from the CSF to the ischemic brain tissue, and the existence of active mechanisms preventing autoreactivity.

## Introduction

Stroke induces an acute sterile inflammatory response in the brain tissue. The cellular participants in this inflammatory process involve activated microglia, monocyte-macrophages, granulocytes, dendritic cells (DCs), and lymphocytes. The pathophysiological role of these inflammatory cells in experimental models of stroke is under study. Knowledge of the inflammatory response in human stroke is limited and mainly focused on the innate immune responses that develop in the acute phase of the stroke. In contrast, adaptive immune responses to stroke would take a longer time to develop. Neuroantigen-specific T cell responses exacerbating the ischemic brain injury have been reported in a transgenic mouse model of experimental ischemia (Jin et al., 2018). In humans, the presence of neuronal- or myelin-derived antigens in draining lymph nodes (palatine tonsils) after stroke was associated with a good or bad prognosis, respectively (Planas et al., 2012).

Antigen-presenting cells (APCs), especially DCs, but also tissue-inflamed resident cells as microglia could bridge innate and adaptive immunity by capturing, processing, and presenting antigens to T cells. The T cell response that issues from the antigenic exposure depends on a variety of molecules and cytokines serving as co-stimuli. Brain ischemia generates sterile inflammation where full activation of these APCs could take place after the release of danger-associated molecular patterns (DAMPs) by dead cells (Gelderblom et al., 2015). Drainage of fluid from the brain to the cervical lymph nodes (cLN) through the cribriform plate was already described (Laman and Weller, 2013). Meningeal lymphatic vessels were identified in the mouse dura mater that drain into deep cLN (Aspelund et al., 2015; Louveau et al., 2015), indicating that this route may transport cells and soluble antigens from the cerebrospinal fluid (CSF) compartment to the draining lymph nodes. The CSF within the ventricle system and the subarachnoid space of the central nervous system (CNS) display immunological functions (Ransohoff and Engelhardt, 2012). Recent studies have provided insights into the mechanisms and dynamics of CSF efflux that may also explain the transport of neural antigens and immune cells out of the brain (Ma et al., 2017, 2019). The presence of sentinel cells scanning CSF-bearing antigens has been described. For instance, DCs are located in the meninges and the choroid plexus under steady-state (Anandasabapathy et al., 2011; Quintana et al., 2015) and DCs circulate through CSF from the brain to draining cLN under neuroinflammatory conditions (Hatterer et al., 2008). Also, effector T cells traffic between leptomeninges and CSF during experimental autoimmune encephalomyelitis (Schläger et al., 2016) and invade the brain through the choroid plexus after stroke in mice as well as in humans (Llovera et al., 2017).

CD4^+^ effector memory T cells constitutively reside in the choroid plexus and participate in an IFN-γ dependent way to control immune cell trafficking through this gate (Baruch et al., 2013; Kunis et al., 2013). It is not known whether in stroke these antigen-experienced T cells respond to neuroantigen-bearing DCs after bystander activation, or whether naïve cLN resident T cells could be primed by active-mature DCs migrating from the CNS. Furthermore, there is also the possibility of T cell priming through indirect antigen presentation mediated by either migratory DC antigen transfer to the cLN resident DCs (Allan et al., 2006) or transfer of peptide-MHCII complexes to lymph node stromal cells (Dubrot et al., 2014). Soluble antigens could also travel out of the brain to draining cLN through the CSF and the glymphatic system (Carare et al., 2008; Albargothy et al., 2018). Whether any resulting T cell priming to brain antigens after ischemic stroke may trigger autoimmune or tolerogenic responses is still unknown (Javidi and Magnus, 2019).

In experimental models of stroke, immune responses to various neural antigens by peripheral T cells and neural-specific B cell responses have been detected in draining lymph nodes and spleen (Ortega et al., 2015). Putative adaptive immune responses against specific brain antigens could have beneficial or detrimental effects on infarct growth and neurological outcome depending on whether the responses were tolerogenic or autoimmune, respectively (Becker et al., 2003, 2011; Gee et al., 2008). In humans, brain-specific short peptides of glial and neuronal cells are released to the CSF and bloodstream after brain ischemia (Urra et al., 2014). The existence of post-stroke infection could provide an adequate environment to give rise to a Th1 autoimmune response to brain-specific antigens (Becker, 2012). Although we did not find relevant antibodies against neural antigens in the blood of stroke patients (Royl et al., 2019), other authors detected transient autoantibodies against the NR2A subunit of the NMDA receptor in stroke patients (Kalev-Zylinska et al., 2013). The presence of these antibodies in serum after a transient ischemic attack (TIA) were poised as predictors of stroke (Weissman et al., 2011), and have been proposed as markers of acute ischemic stroke (Dambinova et al., 2012). However, the existence of a significant neuroantigen-specific adaptive immune response in stroke patients remains to be elucidated.

In this study, we tested whether the presence of brain-derived antigens in the periphery after human ischemic stroke would trigger T cell priming. Pathways for infiltration of activated T cells to the ischemic tissue were explored in post-mortem autopsies. We also evaluated whether the neuronal or myelin origin of neural peptides could elicit a different T helper response and affect the functional outcome in stroke patients.

## Materials and Methods

### Patients

A prospective cohort of 24 subjects, including patients with ischemic stroke (*n* = 13) admitted at the Stroke Unit of the Hospital Clínic de Barcelona, and healthy control donors (*n* = 11). Patients were part of a prospective study (PI12/01437) aimed at measuring the presence of autoimmune responses after stroke, in which all blood samples and neuroimaging were available, and an age-matched control subject group. Table 1 summarizes the demographic and clinical evolution of these patients. Blood withdrawal in sodium-heparin vacutainers was performed on admission, on day 5, and day 90 after stroke. Blood cell counts at admission and day ONE post-stroke are shown in Supplementary Table S1. The study was approved by the local ethics committee (CEIm, Hospital Clinic), and participants or their relatives signed a written informed consent according to the Declaration of Helsinki. Stroke severity was assessed within 12 h of clinical onset, and daily until discharge, with the use of the National Institutes of Health Stroke Scale (NIHSS). The functional outcome was measured at day 90 with the use of the modified Rankin scale (mRS). Certified neurologists performed the evaluations in a blinded way to research laboratory data. An mRS score of 0–2 was defined as a good outcome. Blood from the middle cerebral artery of a patient suffering stroke and subjected to mechanical thrombectomy was obtained according to prospective ethics approved project nested in the CHOICE clinical trial (NCT03876119).

**Table 1:** Demographics and clinical evolution of the patients in the study.

	Controls	Patients	*p*
*N*	10	13
Age, years, mean (SD)	70 (9)	70 (13)	0.843
Sex, male/female (%)	18/82	38/60	0.386
Proximal occlusion (%)		61.5	
Acute revascularization therapy (%)			
None		23	
i.v.		62	
Endovascular		15	
NIHSS score, median (IQR)			
Admission		11(5–15)	
Day 5		2 (0–13)	
Day 90		0.5 (0–5)	
Stroke-associated infection (%)		38.5	
mRS at day 90, median (IQR)		2 (0–4)	

### Post Mortem Study of the Brain of Stroke Patients

We studied the post-mortem brain tissue of nine patients with lethal ischemic brain infarction deceased at the Stroke Unit of the Hospital Clínic de Barcelona. Additional approval of the Ethics Committee of the hospital was obtained for this substudy. Written consent was requested from their families for tissue donation for research purposes at the Neurological Tissue Bank (NTB) of the Biobank-Hospital Clinic-Institut d’Investigacions Biomèdiques August Pi i Sunyer (IDIBAPS). Brain tissue and CSF from the third ventricle for flow cytometry studies were obtained from three cases. Also, brain sections were obtained from six different patients. Table 2 summarizes the clinical data of stroke patients. All patients were women, except for one man, aged between 57 and 89 years old (mean 78 years), and had radiologically confirmed ischemic infarcts. The elapsed time from stroke onset to death ranged between 1 and 140 days. The time from death to tissue processing lasted from 2 h to 8 h. An expert neuropathologist dissected the ischemic core (ipsilateral sample), the contralateral non-ischemic tissue, and collected the CSF from the third ventricle. For the flow cytometry study, small brain tissue portions measuring approximately 20 mm × 20 mm × 10 mm were kept at 4°C in complete RPMI 1640 (RPMI 1640, 2 mM glutamine, 100 U/ml penicillin, 100 μg/ml streptomycin, 10% FBS) until processing for tissue dissociation and flow cytometry analysis. Brain tissue portions were sliced with a scalpel and homogenized with 5 ml of Hanks balanced salt solution (HBSS) without Ca^2+^, Mg^2+^ and supplemented with collagenase IV (50 U/ml, Sigma) and DNase I (150 U/ml, Sigma) in a C-tube (Miltenyi Biotech) by using the GentleMACS dissociator with heaters (Miltenyi biotech) and running the program 37C_ABDK_1. Cells were filtered in a 70-μm cell strainer (Falcon) and washed with HBSS with Ca^2+^ and Mg^2+^. Myelin was removed by gradient centrifugation with 30% isotonic Percoll (GEHealthcare), and cells were washed twice in FACS buffer (PBS, 2 mM EDTA, 2% FBS, 0.01% sodium azide). Finally, cells were suspended in 0.5 ml of FACS buffer with human FcR blocking reagent (1:10 dilution, Miltenyi Biotech) and aliquots of 0.05 ml were stained with live/dead Aqua (1:1,000, Molecular Probes), and the combination of the next antibodies: anti-CD45 (V450, 1:100, clone HI30, Tonbo Biosciences; PerCP, 1:25, clone 2D1, BD Biosciences), anti-CD11b (APC-Cy7, 1:100, clone ICRF44, BioLegend), anti-CD3 (APC, 1:25, clone HIT3a, BD Biosciences), anti-CD4 (PE, 1:25, clone RPA-T4, BD Biosciences), anti-CD8 (PerCP, 1:25, clone SK1, BD Biosciences), anti-CD69 (1:100, clone FN50, BD Biosciences). Isotype controls were performed by using the following antibodies: Mouse IgG1 (V450, 1:100, clone MOPC-21, BD Biosciences; PerCP, 1:25, clone X40, BD Biosciences; PE, 1:25, clone MOPC-21, BD Biosciences; PE-Cy7, 1:100, clone MOPC-21, BD Biosciences; APC-Cy7, 1:100, clone MOPC-21, BioLegend), mouse IgG2a (APC, 1:25, clone G155-178, BD Biosciences). Data were acquired on a FACSCanto II flow cytometer (BD biosciences) with Diva software (BD Biosciences) and analyzed with FlowJo (v10, BD Biosciences).

**Table 2:** Clinical data of the post-mortem stroke patients.

Case	FC1	FC2	FC3	IHC1	IHC2	IHC3	IHC4	IHC5	IHC6
Age	86	57	81	88	79	86	89	74	63
Gender	Woman	Woman	Woman	Woman	Man	Woman	Woman	Woman	Woman
Admission NIHSS	20	11	13	19	4	1	9	35	20
Vascular territory	Left MCA infarct	Right PICA infarct	Left MCA infarct and right MCA recurrent infarct	Right MCA infarct and left MCA recurrence	Left MCA infarct	Vertebro-basilar infarct	Vertebro-basilar infarct	Left MCA infarct	Right MCA infarct
Intracranial occlusion site	Left M1	Right vertebral artery	Left M2	Right and left M1	Left M2	Basilar artery	No vessel imaging	No vessel imaging	Right M1
Acute revascularization therapy	None	None	None	Yes	None	None	None	None	Yes
HT	No	No	No	HI2	PH2	No	No	No	No
Etiology	Cardio-embolic	Other: dissection	Undeter-mined	Cardio-embolic	Lerge vessel disease	Cardio-embolic	Cardio-embolic	Undeter-mined	Undetermined
Exitus (days after onset)	6	140	1	5	5	3	1	18	1
Exitus to necropsy timelapse	2 h + 20 min	7 h	8 h	5 h + 45 min	4 h + 35 min	3 h	3 h	3 h	2 h
Acute infection	Yes, tracheo-bronquitis	Yes, pneumonia	No	No	No	Yes, pneumonia	No	No	No

*FC, Flow cytometry; IHC, immunohistochemistry; MCA, middle cerebral artery; M1, first segment of the MCA; M2, the second segment of the MCA; HT, hemorrhagic transformation; HI2, hemorrhagic infarct type 2; PH2, parenchymal hematoma type 2*.

For immunohistochemistry, we studied T cells in 5-μm paraffin brain sections of the core of the lesion, the ipsilesional periventricular region, and the meninges, when available. We used the anti-CD3 antibody (# ab5690 from Abcam; diluted 1:50) after antigen retrieval with citrate at pH 6 at 110°C for 15 min in the autoclave. Sections were processed with Dako REAL EnVision Detection System, Peroxidase/DAB, Rabbit/Mouse, HRP (# K5007, Dako). Finally, the sections were lightly counterstained with hematoxylin and examined under the optical microscope. The number of cells per area was counted in a large region of interest (ROI area = 35.65 mm^2^) using the 20× objective of a microscope (Olympus BX51) with a motorized stage (Prior Pro Scan II) and equipped with a digital camera (Olympus DP71). Cell counting was performed using stereology software (Visiopharm Integrator System, newCAST™ version 3.2.4.0, Visiopharm, Hoersholm, Denmark). Values are expressed as the number of CD3^+^ cells per mm^2^.

### Isolation of Peripheral Blood Mononuclear Cells (PBMCs) and *in vitro* Stimulation

PBMCs from stroke patients (*n* = 13) at day 0 (admission day), 5, and 90 post-ischemia and from age-matched control donors (*n* = 10) were obtained after gradient centrifugation of blood with Ficoll-Paque (GE-Healthcare) at 500 xg for 35 min at room temperature. PBMCs were resuspended in complete culture medium (RPMI 1640-Glutamax, 100 U/ml penicillin, 100 μg/ml streptomycin, 10 mM HEPES, 1 mM sodium pyruvate, 50 μM β-mercaptoethanol, 5% ABS serum) at 10^7^ cells/ml and plated in 96 well/plate U bottom at 2 × 10^6^ cells/well. Reconstituted peptides were added to cell suspension (0.6 nmol or its equivalent of 1 μg of peptide/ml) and incubated at 37°C and 5% CO_2_ for 24 h. Basal values were obtained from PBMC of the same patients by incubating some of the cells under the same conditions but in the absence of peptides in the same experiment. Polyclonal T cell reactivity was explored by stimulation with 50 ng/ml PMA, 1 μM ionomycin. The last 6 h BD GolgiStop and BD GolgiPlug (BD Biosciences) were added to the culture according to the manufacturer’s instructions.

### Selection and Synthesis of Peptides From Four Different Neural Proteins

We analyzed two myelin-derived proteins (MOG and MBP), and two neuronal proteins (NMDA subunit 2A named as NR2A, and the neuronal cytoskeletal proteinMAP2). Peptides derived from the two myelin proteins were purchased from Miltenyi Biotech (PepTivator MBP isoform-research grade human 130-097-287 Miltenyi Biotech and Peptivator MOG research-grade human 130-096-770 Miltenyi Biotech). Both are a pool of 15-mer peptides with overlapping sequences of 11 amino acids (aa) and covering the sequence of the entire protein. The NR2A and MAP2 peptides were selected *in silico* based on immunogenicity and the ability to bind to HLA. For NR2A (GI:14285603) only the first 150 aa of the N-terminal part was considered for antigenic properties after discarding the 22 aa of signal peptide based on previous data showing immune-response in stroke patients to this extracellular region (Weissman et al., 2011; Dambinova et al., 2012). For MAP2 (GI:215274255), the aa sequence 1529–1827 was selected because it showed a 100% identity for all isoforms. The selected protein sequences were introduced in *in silico* tools to look for MHCII binding regions: RANKPEP^1^, SYFPEITHI^2^, SVMHC^2^, NetMHCIIpan3.0^3^, and IEDB^4^. The selected peptides (see Table 3) for NR2A and MAP2 were synthesized and purchased from ProImmune Inc. and resuspended in PBS after checking peptide polarity and isoelectric point (pI), according to the supplier.

**Table 3:** List of peptides selected for immunogenicity to NR2A and MAP2.

Peptide	Start	End	Length	Sequence
NR2A11	11	28	18	ALNIAVMLGHSHDVTERE
NR2A38	38	55	18	AAGLPLDVNVVALLMNRT
NR2A58	58	77	20	KSLITHVCDLMSGARIHGLV
NR2A87	87	110	24	VAQMLDFISSHTFVPILGIHGGAS
NR2A145	145	159	15	WHVFSLVTTIFPGYR
NR2A152	152	166	15	TTIFPGYREFISFVK
MAP13	13	27	15	TSKCGSLKNIRHRPG
MAP16	16	30	15	CGSLKNIRHRPGGGR
MAP33	33	47	15	IESVKLDFKEKAQAK
MAP54	54	68	15	AHHVPGGGNVKIDSQ
MAP67	67	81	15	SQKLNFREHAKARVD
MAP76	76	93	18	AKARVDHGAEIITQSPGR
MAP100	100	114	15	RRLSNVSSSGSINLL

### Intracellular Cytokine Staining

PBMCs were washed with FACS buffer and stained for cell viability with the fixable live/dead Aqua staining kit (Molecular Probes) in the presence of Fc-R blocking reagent (Miltenyi biotech), and the antibodies against the surface markers anti-CD3-APC, CD45-V450 and anti-CD11b-APC/Cy7 indicated above. Surface staining was carried out for 25 min at 4°C. Cells were washed, and intracellular staining was carried out with BD cytofix-cytoperm kit (BD Biosciences). Cells were fixed with Fix-Perm (BD Biosciences) for 20 min at room temperature and permeabilized with Perm-Wash (BD Biosciences) for 30 min at room temperature. Cells were stained with two combinations of antibodies diluted in Perm-Wash: anti-IFN-γ (PE, 1:100, clone 4S.B3, BD Biosciences), anti-IL-4 (FITC, 1:100, clone MP4-25D2, BD Biosciences), and anti-IL-17A (PE/Cy7, 1:100, clone BL168, BioLegend) or anti-TNF-α (AlexaFluor-488, 1:100, clone MAb11, BD Biosciences) and anti-IL-10 (PE, 1:30, clone JES3-9D7, BD Biosciences). Data were acquired on a FACSCanto II flow cytometer (BD Biosciences) with Diva software (BD Biosciences) and analyzed with FlowJo (v10, BD Biosciences).

### Statistical Analysis

Comparisons between patients and controls were adjusted for age and sex. Continuous variables were reported as mean ± standard deviation or median with interquartile ranges, as indicated in each figure legend. For continuous data, the student’s *t*-test, one-way analysis of variance, Mann-Whitney, or Kruskal-Wallis were applied. Categorical variables were analyzed with the Chi-square test or Fisher exact tests. Ordinal regression models were used to assess the numbers of cytokine-producing CD3^+^ lymphocytes on functional outcome at 90 days. All tests were performed using the SPSS software version 20.0 (SPSS Inc.). *P* values < 0.05 were considered statistically significant.

## Results

### Activated CD3^+^ Lymphocytes in Postmortem Brain of Stroke Patients

**Figure 1:**
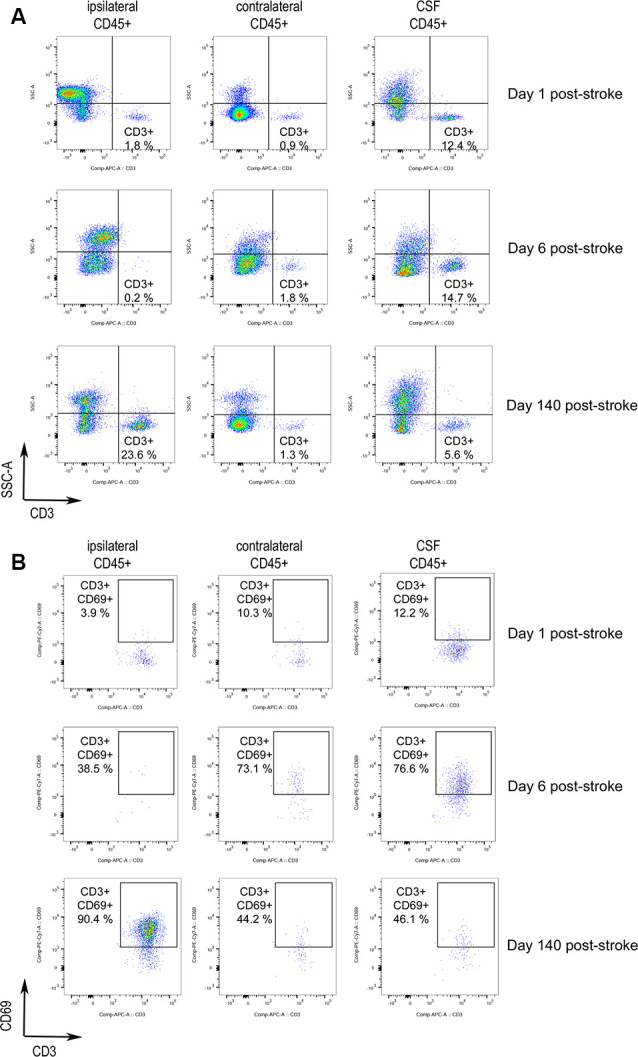
Activated T cells in human stroke brain tissue at the chronic phase. Post-mortem brain tissue was obtained from three stroke patients FC3, FC1, and FC2 deceased 1, 6, and 140 days post-stroke, respectively (Table 2). **(A)** Dot-plots of flow-cytometry data of the brain ipsilesional and contralateral area and the cerebrospinal fluid (CSF) 3rd ventricle. CD3^+^ SSClow lymphocytes are identified. CD3^+^ lymphocytes were gated from previous live single CD45+ cells showed in Supplementary Figure S1B. **(B)** Analysis of CD3^+^ SSClow cells for the expression of the lymphocyte activation marker CD69 showed initial activation in the CSF followed by later T cell activation in the ischemic brain hemisphere.

The brain of three patients deceased 1, 6, and 140 days after stroke onset was analyzed by flow cytometry to characterize the presence of infiltrating lymphocytes (see Table 2 for clinical features). The brain samples corresponded to the ischemic core (ipsilesional area), the corresponding contralesional area, and the CSF obtained from the 3rd ventricle. Supplementary Figure S1 shows the strategy of gating and the identification of live leukocytes (Supplementary Figure S1A), the isotype controls (Supplementary Figure S1B), and dot-plots showing live leukocytes (CD45^+^ cells) for all three patients (Supplementary Figure S1C). Of note, the ipsilesional sample corresponding to 140 days after stroke onset showed a remarkable presence of T cells (Figure 1A), and the majority of the CD3^+^ lymphocytes were activated (CD69^+^, Figure 1B). Notably, at day 140 post-stroke, an important percentage of the CD3^+^ lymphocytes were CD8^+^ (61% CD8^+^ vs. 25% CD4^+^ T cells; Supplementary Figure S2). Post-mortem ipsilesional samples of the patients deceased on day 1 and day 6 post-stroke presented few parenchymal infiltrated T cells with no activation (CD69^+^ T cells).

Notably, the presence of T lymphocytes in the CSF of ventricles was detected in all patients. These T cells were predominantly CD8^+^ (Supplementary Figure S2), e.g., 65% CD8^+^ vs. 25% CD4^+^ T cells for the patient deceased at day 1 post-stroke. T lymphocytes did not show an activated phenotype in the patient deceased on day 1, but CD3^+^ CD69^+^ lymphocytes were observed in the CSF of ventricles of the patient deceased at day 6 post-stroke. The presence of activated T cells in the CSF of the third ventricle pointed to a possible route of T cell entry from the periphery. In support of this possibility, CSF T cells were mainly CD8^+^, as seen in the injured brain parenchyma of the patient deceased 140 days post-stroke. In contrast, CD4^+^ T cells are the majority in the circulation. Furthermore, in the case in which we could analyze blood obtained from the middle cerebral artery during mechanical thrombectomy, the CD4/CD8 ratio of cerebral arterial blood was similar to that of the blood obtained by venipuncture (1.4–1.8).

Activated CD3^+^ CD69^+^ lymphocytes were observed in the CSF mainly at day 6 post-stroke, and in the injured brain parenchyma at day 140 post-stroke (Figures 1A,B). These observations suggest the possibility that naïve T cells primed with neural or glial antigens in the periphery could be activated in the brain parenchyma by its cognate antigen.

**Figure 2:**
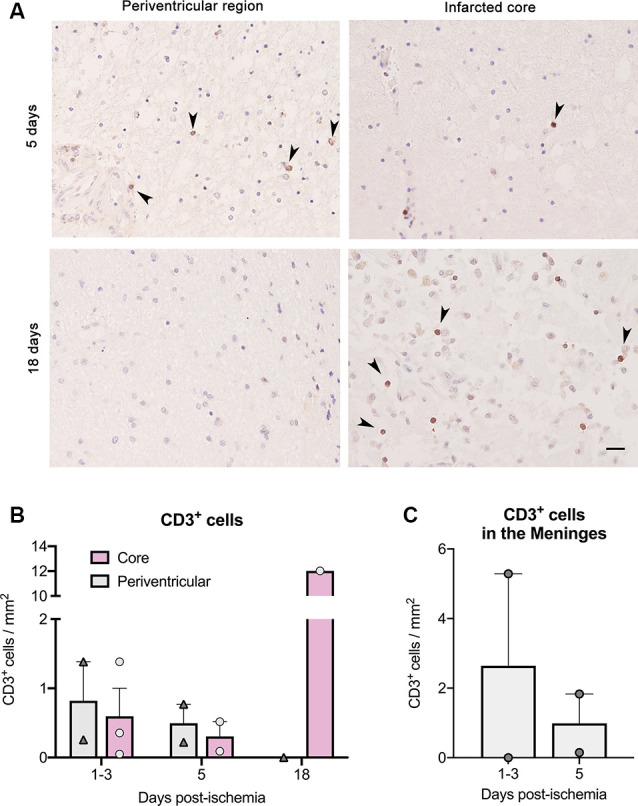
T cell counts after immunohistochemistry in brain samples of six ischemic stroke cases deceased between day 1 and day 18 post-stroke. Features of the patients (IHC1 to IHC6) are shown in Table 2. **(A)** CD3^+^ T cells (dark brown) in paraffin brain sections counterstained with hematoxylin in the core of the lesion and at the periventricular region for patients deceased at day 5 and day 18 post-stroke. Arrowheads indicate CD3^+^ cells. Scale bar: 25 μm. **(B)** For illustration of quantification, patients were grouped according to death 1–3 days post-stroke (*n* = 3), 5 days post-stroke (*n* = 2), and 18 days post-stroke (*n* = 1). **(C)** CD3^+^ cell counts in the meninges of patients where this tissue was available.

We also detected the presence of CD3^+^ T cells in brain sections of another group of six stroke patients (Figure 2). T cells were more abundant in the lesion core of the patient deceased 18 days post-stroke than in the patients deceased at earlier time points (Figures 2A,B). In the patients deceased from 1 to 5 days post-stroke, we observed CD3^+^ T cells in the periventricular region, but T cells were not seen in this location in the patient deceased at day 18 after stroke onset (Figures 2A,B). We also detected T cells in the meninges in some of the patients deceased during the first days post-stroke but meningeal tissue was not available for the patient that died 18 days post-stroke (Figure 2C).

### Neural Antigen-Specific T-Cell Response After Stroke

**Figure 3:**
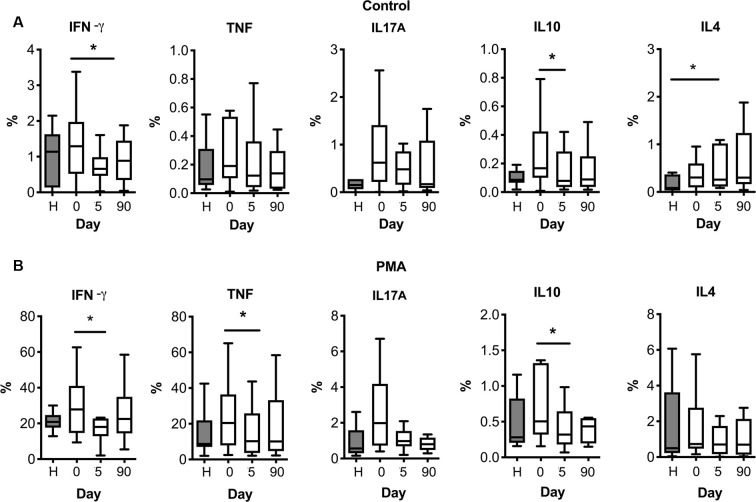
T cell cytokine production after stroke in basal **(A)** or polyclonal challenge **(B)**. PBMC from healthy donors (H) or stroke patients at different days post-ictus (0, on admission; 5, day 5 post-stroke; 90, day 90 post-stroke) were *in vitro* cultured for 24 h in basal conditions **(A)** or stimulated in presence of 50 ng/ml PMA and 1 μM ionomycin **(B)**. Percentages of live CD3^+^ lymphocytes producing pro-inflammatory cytokines (IFN-γ, TNF-α, or IL-17A) or anti-inflammatory cytokines (IL-10 or IL-4) were quantified based on the strategy of gating shown in Supplementary Figure S3 (one-way analysis of variance, *p < 0.05).

We then investigated whether lymphocytes could be primed against brain antigens in the periphery after stroke. We isolated PBMCs from stroke patients at different time points: the day of admission (day 0), 5, and 90 days after stroke. PBMCs from healthy age-matched subjects were included in the study (Table 1). Isolated PBMCs were cultured for 24 h with different neural antigens, and cytokine production was analyzed by flow cytometric intracellular cytokine staining (see Supplementary Figure S3 for gating strategy). Percentages of CD3^+^ lymphocytes producing IFN-γ, TNF-α, IL-10 or IL-17A in basal conditions (Figure 3A) tended to be higher in stroke patients on admission compared to age-matched healthy donors, but differences did not reach statistical significance (admission median (IQR) 1.29 (0.52–1.97) vs. healthy 1.14 (0.13–1.63) for IFN-γ; admission 0.19 (0.10–0.53) vs. healthy 0.10 (0.06–0.31) for TNF-α; admission 0.17 (0.10–0.42) vs. healthy 0.09 (0.06–0.15) for IL-10; admission 0.62 (0.22–1.41) vs. healthy 0.15 (0.06–0.27) for IL-17A). However, the number of T cells producing either pro-inflammatory cytokine IFN-γ or anti-inflammatory cytokine IL-10 significantly decreased at day 5 post-stroke [median (IQR) 0.65 (0.46–0.98)] vs. admission day. On the contrary, the percentage of T cells producing IL-4 significantly increased over time (Figure 3A). Polyclonal stimulation with PMA-Ionomycin also showed higher percentages of CD3^+^ lymphocytes producing IFN-γ, TNF-α, or IL-10 at admission compared to day 5 after stroke (Figure 3B).

**Figure 4:**
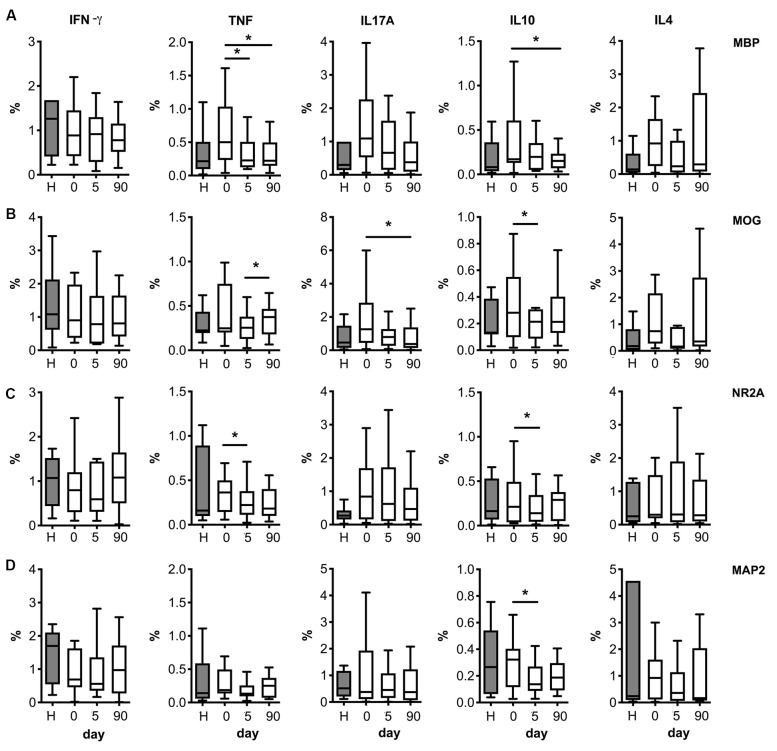
Neural and myelin peptide stimulation of T cells from stroke patients. Cytokine production by T cells co-stimulated with myelin related proteins [MBP peptides **(A)** or MOG peptides **(B)**] or neural derived peptides [NR2A **(C)**, or MAP2 **(D)**]. PBMC from healthy donors (H) or stroke patients at different days post-ictus (0, on admission; 5, day 5 post-stroke; 90, day 90 post-stroke) were *in vitro* cultured for 24 h in presence of myelin or neural peptides and percentages of live CD3+ lymphocytes producing pro-inflammatory cytokines (IFN-γ, TNF-α, or IL-17A) or anti-inflammatory cytokines (IL-10 or IL-4) was quantified after following the strategy of gating shown in Supplementary Figure S3 (one-way analysis of variance, *p < 0.05).

Following stimulation, with either myelin antigens (MBP and MOG, Figures 4A,B) or neural peptides (NR2A, MAP2; Figures 4C,D) the number of T cells producing IL-10 was higher on admission compared to post-stroke day 5. MBP peptides also increased the percentage of T cells producing TNF-α on admission vs. later time points (Figure 4A). A similar effect was induced by MOG antigens on the number of T cells producing IL-17A (Figure 4B).

**Figure 5:**
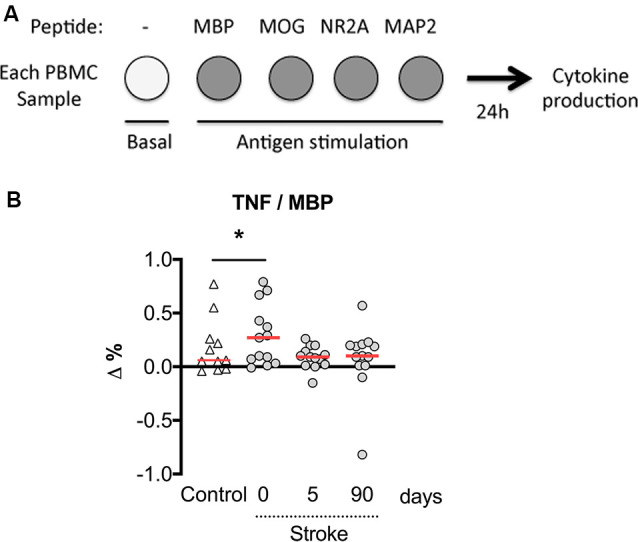
The net percentage of CD3^+^ lymphocytes producing TNF-α with MBP peptides stimulation over basal production. **(A)** Experimental design showing that PBMCs were untreated or exposed to neural peptides for measures of cytokine production at 24 h. **(B)** The numbers of T cells with basal production of TNF-α were subtracted from the numbers of MPB peptide-stimulated TNF-α producing T cells (Δ%). On admission, stroke patients presented a higher percentage of T cells responding to MBP peptides by producing TNF-α. One-way analysis of variance, *p < 0.05.

We then calculated the net percentage of cytokine-producing CD3^+^ lymphocytes in response to myelin or neural peptides after subtraction of the basal cytokine production in the absence of antigen stimulation. Notably, the net production of TNF-α by CD3^+^ lymphocytes in response to the challenge of MBP peptides was higher in patients at admission compared with post-stroke day 5 (Figure 5). In contrast, we did not observe a net production of IL-10 in response to myelin or neural peptides, or IL-17A in response to MOG peptides (not shown). In summary, a small and transient neural-antigen specific inflammatory response by T cells was only observed against the myelin peptides coming from MBP protein in the acute phase of the stroke.

### Cytokine Production by T Cells and Stroke Outcomes

**Figure 6:**
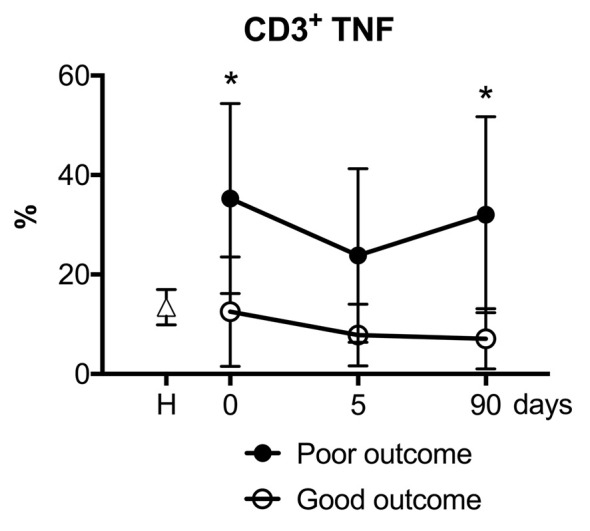
Patients with good functional outcomes presented fewer TNF-α producing CD3+ lymphocytes. Stroke patients with a good functional outcome (open circles) at 90 days measured with modified Rankin scale (mRS 0–2) showed a lower percentage of polyclonal activated CD3+ lymphocytes that produced pro-inflammatory cytokine TNF-α compared to stroke patients with poor outcome (filled circles). Filled square, healthy control donors. One-way analysis of variance, *p < 0.05.

We then explored whether the presence of cytokine-producing T cells in this cohort of stroke patients was associated with the functional outcome as assessed by the mRS. In an exploratory analysis, the numbers of reactive CD3^+^ lymphocytes producing TNF-α was significantly higher in the subgroup of patients with poor outcome at 90 days after stroke (Figure 6). The production of IL-10 or IL-4 by CD3^+^ lymphocytes in the first days of acute stroke was not associated with the functional outcome at 90 days. However, the non-specific production in response to PMA of pro-inflammatory TNF-α by CD3+ lymphocytes at admission was related to poor stroke outcomes at 90 days even after adjusting for baseline stroke severity (OR 1.13, 95% CI 1.01–1.27, *p* = 0.041).

## Discussion

Activated T cells were observed in the brain of patients several days after stroke. The presence of T lymphocytes was observed by flow cytometry in the CSF of the ventricles in two patients deceased at 1 and 6 days after stroke onset, but only T cells of the latter patient displayed an activated phenotype, whereas T cells invaded the ischemic tissue of the patient deceased 140 days after stroke. The presence of T cells in the ischemic parenchyma was confirmed by immunohistochemistry in a patient deceased 18 days after stroke whereas T cells were less abundant in patients deceased from 1 to 5 days post-stroke. Low numbers of T cells were detected in the hemisphere contralateral to the ischemic lesion that may be explained by some degree of circulating leukocyte contamination, given that human brain samples were obtained from non-perfused tissues. Likewise, T cell numbers were low in the ipsilesional brain hemisphere during the first week after stroke onset, but their presence was notorious at 18 days and 4 months. The nature of the human study did not allow us to follow the time course of T cell infiltration or even to have samples at pre-defined delays from a stroke. However, the CSF could be one plausible route of T cell entry because T lymphocytes were found in the ventricles, in two patients deceased during the first-week post-stroke, whereas the patient deceased at day 140 post-stroke showed less CD3^+^ cells in the CSF of ventricles. In experimental autoimmune encephalomyelitis, where brain auto-reactive T cells immigrate to the CNS, trafficking of effector T-cells was observed between leptomeninges and the CSF (Schläger et al., 2016). In a stroke, besides leukocyte extravasation from the brain parenchymal vessels, both meningeal and choroid plexus T cell invasion routes have been reported (Llovera et al., 2017). This latter route is also plausible in humans according to our results since the CSF obtained from the ventricles of deceased stroke patients was enriched in T lymphocytes.

A distinctive feature of T cells infiltrating the ischemic brain is the CD4 vs. CD8 lymphocyte ratio, which is lower than that observed in the blood, even for stroke patients. We checked whether this imbalance in favor of CD8 T cells instead of CD4 T cells could have occurred locally in the cerebral vasculature of stroke patients by comparing blood from the middle cerebral artery obtained during a mechanical thrombectomy therapy with venipuncture blood of stroke patients. Nonetheless, the CD4/CD8 ratio of cerebral arterial blood was like venipuncture blood. Also, a human neuropathological study of brain tissue lesions reported that the primary infiltrating lymphocytes in ischemic infarct lesions were CD8^+^ T cells (Zrzavy et al., 2018). Although the CD8 number was very low compared to other CNS inflammatory diseases (Zrzavy et al., 2018), the time points of study after stroke could be critical, given the dynamic nature of leukocyte infiltration. Altogether, our data pointed to the presence of a unique population of activated T cells in the brain of stroke patients at the chronic phase that was not observed in the acute or subacute phase.

Roles of T cells have been mainly investigated in the acute phase of ischemia. They include increasing neuroinflammation (Yilmaz et al., 2006), thromboinflammation (Kleinschnitz et al., 2011), and preventing hemorrhagic transformation (Salas-Perdomo et al., 2018) or secondary damage in the case of regulatory T cells (Liesz et al., 2009). An antigen-dependent neurotoxic effect mediated by perforin release was described for infiltrating CD8^+^ T cells in the first 2 weeks after experimental stroke (Mracsko et al., 2014). Moreover, MOG-specific T cell proliferation with increased neurological deficits and infarction volumes has been reported at day 4 after brain ischemia in mice (Jin et al., 2018). T cell clonal expansion following a stroke in an experimental model of permanent ischemia was observed in the brain, before lymphoid tissue, from 7 days post-infarct when most detrimental effects of post-ischemic neuroinflammation have already occurred (Liesz et al., 2013). Although the underlying mechanisms are not well understood and the contribution to acute brain damage is unlikely, possibly glial and neuronal antigen-dependent responses may affect long-term stroke complications such as depression, cognitive impairment, and dementia. Nonetheless, experimental studies showed that an increase of CNS antigen-specific T cell responses did not worsen long-term functional outcomes (Römer et al., 2015). Accumulation of regulatory T cells has been observed in the brain from 15 to 30 days after stroke in mice (Stubbe et al., 2013). Furthermore, a role for T cells in functional recovery has been suggested (Selvaraj and Stowe, 2017).

Whether brain infiltrating T cells are neuroantigen-experienced lymphocytes is a long-debated question. A collection of data pointed to the drainage of brain antigens through the CSF to the periphery. After a stroke, the presence of neural antigens was observed in draining lymph nodes close to activated CD3^+^ CD69^+^ lymphocytes (Planas et al., 2012). The existence of an adaptive immune response to brain antigens following brain injury has been explored (Becker et al., 2011, 2016). Here, we addressed this question by checking the peripheral T cell response to different glial and neuronal antigens in PBMCs obtained at sequential times in stroke patients. We found that blood T lymphocytes from stroke patients were sensitized to myelin peptides of MBP on the day of admission. However, this response was transient and disappeared after the acute phase of the stroke. Previous studies showed increases of MBP antigens (along with neuron-specific enolase and S100-β) in the serum of stroke patients in the first 24 h after stroke. MBP-reactive T cells may exist in the circulation (Mundt et al., 2019). The pro-inflammatory environment of the acute phase of a stroke may provide the co-stimulatory signals necessary for T cell activation. *In vivo*, peripheral T cells may encounter myelin antigens after stroke due to blood-brain barrier breakdown or access of myelin peptides to the draining lymph nodes. An association of higher MBP peak concentrations with larger lesion volumes and poor outcomes was demonstrated (Jauch et al., 2006). Both experimental and clinical studies evidenced that Th1 responses to MBP are associated with worse neurological outcome after stroke when infection occurred, and this kind of immune response could aggravate the neurologic deficits (Becker et al., 2011).

MBP induced TNF-α production by T cells and patients with a higher percentage of these T cells presented poorer outcomes. However, this difference was not significant when accounting for the severity of stroke. On the contrary, TNF-α production in response to PMA predicted poor functional outcomes even after accounting for the main prognostic factor, which is the baseline severity of the stroke. According to general observations of a concomitant increase in the number of inflammatory cells and cytokine production at the onset of stroke, the percentage of blood T cells producing IFN-γ was higher in stroke patients at admission and fell in the following days. This situation of T cell inflammation at admission was observed more clearly when polyclonally restimulated lymphocytes produced both IFN-γ and TNF-α. A detrimental pathophysiological role for T cells producing IFN-γ in the early stage of ischemic stroke was previously shown (Yilmaz et al., 2006). Even though inflammatory cells other than T lymphocytes producing IFN-γ or TNF-α could participate in stroke-derived neurological deficits, the association of a higher percentage of T lymphocytes producing TNF-α with poorer functional outcomes at 90 days highlights the negative relevance of an inflammatory lymphocyte-mediated response in human stroke.

IL-10 producing T lymphocytes also increased after stroke onset, suggesting that stroke generated a new homeostatic level to offset the previous inflammatory context, and this counterbalance is set within the T cell compartment. A failure in this system could promote worse neurological outcomes since lower IL-10 plasma levels were associated with clinical worsening in ischemic stroke patients (Vila et al., 2003). Interestingly, T lymphocytes upregulated the production of the anti-inflammatory cytokine, IL-4, with different kinetics than IL-10. Whereas IL-10 producing lymphocytes were upregulated at admission, the proportion of T lymphocytes producing IL-4 increased all over the 90 days from the onset of stroke. This Th2 cytokine induces macrophage M2 polarization suggesting that brain infiltrating lymphocytes in the chronic phase of ischemia may contribute to skew microglia/macrophages functions towards an M2 phenotype and participate by this way in brain repair.

There are some limitations in the present study, the most significant one being the modest number of patients. In the case of blood studies, this is balanced by the standardized protocol of repeated sample measurements, which allowed for comparing the cellular responses in different time-points after stroke. However, this was not possible in the case of post-mortem brain samples, whose timing depended on the natural history of the disease. Another limitation is that for both myelin proteins (MBP and MOG), the peptides covered the full length of the proteins. However, NR2A has 1,464 aa, and MAP2 present different isoforms with isoform 1 of 1,827 aa. For this reason, we limited the MAP2 and NR2A peptides to regions that were considered more antigenic. Overall, the lymphocyte response to neural antigens was poor. Finally, we cannot exclude that some of the changes in cytokine T cell production in the acute phase of stroke could be related to stress factors other than brain damage and related to hospitalization. Further studies will need to control this possibility including hospitalized patients devoid of brain damage.

Despite these limitations, we were able to identify increased production of both proinflammatory TNF-α, and anti-inflammatory IL-10 in response to neural peptides in lymphocytes of stroke patients at admission vs. day 5 post-stroke. The magnitude of lymphocyte TNF-α production in response to MBP during the acute phase of stroke was associated with poor stroke outcomes. However, we did not detect specific responses of peripheral T lymphocytes to neural antigens in the subacute and chronic phase of stroke, suggesting that generally the development of sustained and potentially damaging autoimmune responses is inhibited after stroke.

## Data Availability Statement

The raw data supporting the conclusions of this article will be made available by the authors, without undue reservation.

## Ethics Statement

The studies involving human participants were reviewed and approved by Comitè d’Ètica d’Investigació (CEIm) Hospital Clínic de Barcelona. The patients/participants provided their written informed consent to participate in this study. For post-mortem brain tissue, written consent was requested from the families for tissue donation for research purposes at the Neurological Tissue Bank (NTB) of the Biobank-Hospital Clinic-Institut d’Investigacions Biomèdiques August Pi i Sunyer (IDIBAPS).

## Author Contributions

FM-M conceived, designed, performed experimental work, analyzed data, and wrote the draft manuscript. FR-J performed experimental work. XU selected the patients, contributed to the conception and design of the study, analyzed data, and revised the manuscript. JP conducted the immunohistochemical study in brain tissue sections. ÁC conceived the study and revised the manuscript. AP conceived the study, analyzed data, and revised the manuscript.

## Conflict of Interest

The authors declare that the research was conducted in the absence of any commercial or financial relationships that could be construed as a potential conflict of interest. The reviewer MG declared a past co-authorship with one of the authors AP to the handling Editor.

## Footnotes

^1^imed.med.ucm.es

^2^www.syfpeithi.de

^3^www.cbs.dtu.dk/services/NetMHCIIpan/

^4^IEDB.org
