# Supplementary material for: Antigen-Dependent T Cell Response to Neural Peptides After Human Ischemic Stroke
Source: Front Cell Neurosci. 2020 Jul 3;14:206. doi: 10.3389/fncel.2020.00206 (PMC7348665; doi:10.3389/fncel.2020.00206)
Supplement: Supplementary file 1 [file Data_Sheet_1.PDF]

Antigen-dependent T cell response to neural peptides after human ischemic stroke

Francesc Miro-Mur, Xabier Urra, Francisca Ruiz-Jaen, Jordi Pedragosa, Angel Chamorro, Anna M. Planas

SUPPLEMENTARY FIGURES & TABLE

Supplementary Figure S1

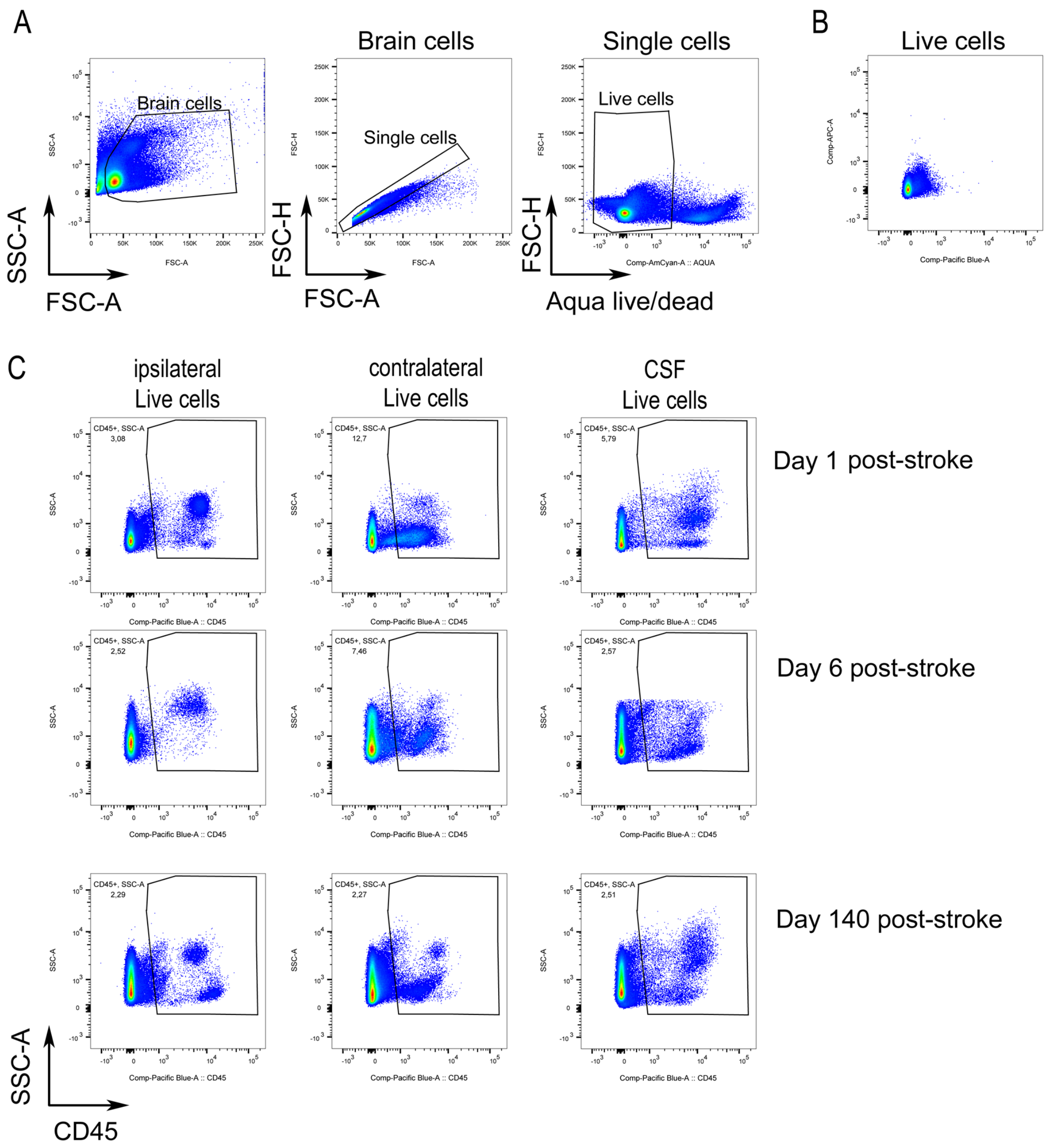

Supplementary Figure S1. Gating strategy to study the presence of CD3+ lymphocytes in the ipsilesional and its contralateral area and the CSF from the 3rd ventricle. A) Images for selection of live single cells of brain biopsies correspond to the IC9 patient which have the longest time-lapse from exitus to necropsy (see table 1). B) Dot-plot of live single cells with the isotype antibody controls used for the gating for $CD45^+$ cells (panel C) and $CD3^+$ cells (Fig. 1). C) $CD45^+$ leukocytes gated from previous live single cells of brain biopsies from patients FC3 (Day 1 post-stroke), FC1 (Day 6 post-stroke) and FC2 (Day 140 post-stroke) (Table 2).

Supplementary Figure S2.

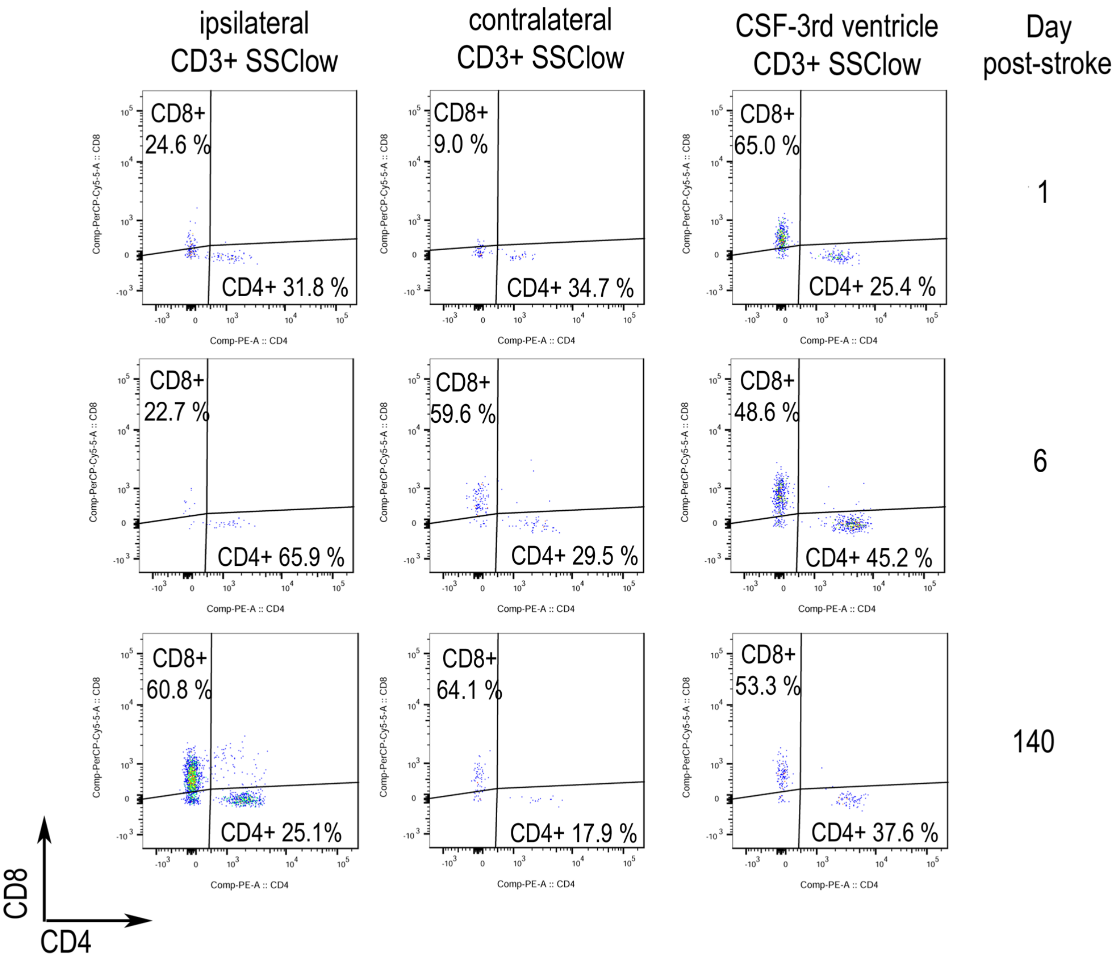

Supplementary Figure S2. Immunophenotype of brain $CD3^+$ cells. Dot-plots of flow-cytometry data of the brain ipsilesional and contralateral area and the CSF 3rd ventricle corresponding to postmortem brain tissue of stroke patients FC3 (day 1 post-stroke), FC1 (day 6 post-stroke) and FC2 (day 140 post-stroke). $CD3^+$ SSClow gate shows the presence of both $CD4^+$ and $CD8^+$ T cells in the ischemic brain tissue at chronic phase as well as in the CSF 3rd ventricle at early time points.

Supplementary Figure S3.

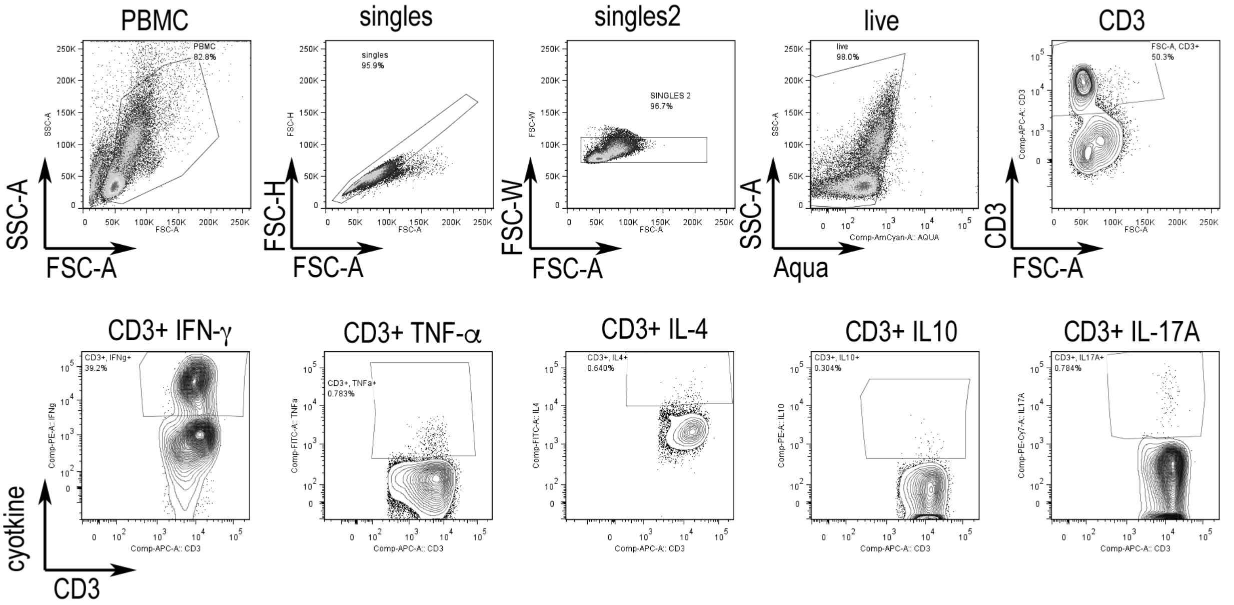

Supplementary Figure S3. Gating strategy of $CD3^+$ cells from PBMC of stroke patients and representative dot-plots of flow cytometric intracellular cytokine staining (FACS-ICCS) for IFN-γ, TNF-α, IL-4, IL-10 and IL-17A inside CD3 lymphocytes.

Supplementary Table S1: Blood leukocyte counts at day 0 and day 1 after stroke.

Day 0:

	**Median**	**Q1**	**Q3**
Leukocytes	8.1	6.9	10.3
Neutrophils	5.1	4.6	7.9
Lymphocytes	1.5	1.1	2.3
Monocytes	0.5	0.5	0.7

Day 1:

	**Median**	**Q1**	**Q3**
Leukocytes	9.4	7.1	10.9
Neutrophils	6.2	5.1	8.8
Lymphocytes	1.5	1.0	2
Monocytes	0.7	0.4	0.9

Values are expressed as the median and Interquartile Range (IQR)
